# Characterization of a Vision-Based Tool for the Investigation of Geometric Characteristics of Ground-Deposited Volcanic Ash

**DOI:** 10.3390/s22249616

**Published:** 2022-12-08

**Authors:** Bruno Andò, Salvatore Baglio, Salvatore Castorina, Alberto Campisi

**Affiliations:** Department of Electrical, Electronics and Computer Engineering (DIEEI), University of Catania, 95125 Catania, Italy

**Keywords:** volcanic ash, vision system, embedded tool, image processing, characterization, robustness analysis

## Abstract

With the support of public authorities and research institutions, volcanic ash fallout and its impact on the safety of people, infrastructure and services are addressed with the aim of defining protocols and instruments for the reliable and effective handling of related emergencies. Most of the solutions proposed in the literature on ash fallout monitoring suffer from high cost and are demanding in terms of installation and maintenance. The approach suggested in this work is based on the use of a low-cost vision embedded system and a dedicated algorithm which automatically processes acquired frames of ground-deposited volcanic ash in order to estimate the main geometric properties of each particle identified in the work area. A complete characterization of the system is presented, along with a robustness analysis of particle shapes, their orientation and their position in the inspected frame. An accuracy of ±40.2 µm (with a 3σ limit) and a resolution of 32.9 µm (in the worst case), over a framed area of 130 mm by 100 mm, were estimated; these values would fulfill the objectives of the application.

## 1. Introduction

Volcanic activity involving the emission of ash clouds into the atmosphere represents a significant hazard to airplane transport and airports on a global scale. The main risk factor for air transport is volcanic ash in the airspace near airports or deposited on airport runways and infrastructure. During an eruption, the ash plume ejected by a volcano can quickly reach the altitudes at which aircrafts fly. When an airplane encounters a volcanic ash cloud, the effects include [1]: malfunction or failure of engines; malfunction of sensors, resulting in unreliable indications and warnings; windscreens rendered partially or completely opaque; smoke, dust and/or toxic chemical contamination of cabin air; erosion of aircraft components; and reduced electronic cooling efficiency. The risks posed by volcanic ash clouds to aircraft in flight have been extensively addressed. For example, in [2], information about reported encounters of aircraft with volcanic ash clouds from 1953 to 2009 is collected and analyzed. The document reports 129 accidents over that period, about 73% of which were due to encounters with volcanic ash, of which about 61% concerned cases in which damage to the structure and/or engines of the aircraft was reported. In about 20% of the examined cases, the reported damage was classified as significant to very serious, and in 7% of cases, the engines stopped during flight. In the period from 1976 to 2009, there were, on average, two events per year related to encounters with volcanic ash clouds, most of which occurred within 24 h of the beginning of the eruption and less than 1000 km from the volcano.

Other problems faced by the aviation sector due to volcanic activity include operational disruptions and physical damage to airports. Concerning airport infrastructure, the main hazard is ashfall. The presence of volcanic ash in the airspace surrounding an airport can reduce visibility for safe takeoff and landing. Volcanic ash accumulates on runways; even a layer of ash a few millimeters thick degrades breaking performance and may lead to runway closures from a few hours to several days. Moreover, volcanic ash may infiltrate electrical systems, interrupt ground services and damage buildings and parked airplanes. In order to resume full operation, the volcanic ash accumulated on airport runways must be removed. A set of recommended procedures for the protection and clean-up of ash-contaminated airports is reported in [3].

An analysis of airport incidents due to volcanic activity in the period of 1944–2006 is well elaborated in [4]. Some airports have been frequently affected by volcanic activity. In particular, the Vincenzo Bellini (Fontanarossa) International Airport, located in Catania, Italy, about 30 km from Mount Etna (the tallest active volcano in Europe), experienced six incidents related to volcanic activity, with the most recent being in 2001 and 2006. The most common reported inconveniences include sky obscuration and difficulties in handling air traffic to and from Catania airport. Other airports that have been affected by the volcanic activity of Etna in recent years are the Sigonella Naval Air Station, located about 38 km from the volcano, and the Reggio Calabria airport, 68 km from Etna.

On a worldwide basis, about 20% of airports affected by volcanic activity are located within 30 km from a volcano, about 30% at a distance of between 30 km and 150 km, about 25% between 150 km and 300 km and the remaining 25% at distances greater than 300 km [5].

The consequences of ash fallout on the operation of airports located close to active volcanoes can be limited by adopting specific countermeasures, including protecting parked aircraft and other equipment, scheduled cleaning of runways, as well as the adoption of dedicated procedures for take-off and landing. However, in order to mitigate the effects of ash fallout, models which are able to predict the dispersion of ash plumes in the atmosphere over time are useful. Several models for ash transportation and dispersion have been developed with the aim of providing aviation authorities with forecasts of ash dispersion in space and time [6,7,8,9]. In particular, ash dispersion models which are also capable of forecasting ash deposition on the ground are of particular interest. The reliability of model predictions is strongly dependent on (i) the quality of wind forecasts [10,11,12], and (ii) the availability of accurate information related to ash size distribution and concentrations in the atmosphere, as investigated in [13,14,15]. In particular, in these works, it is demonstrated that the accuracy of forecasts of the dispersion of ash particles in the atmosphere is significantly affected by the particle size distribution used to feed the model, due to the relationship between the fall velocity of modeled particles and particle size. In fact, larger particles (diameter ≥ 100 µm) fall five orders of magnitude faster than smaller particles (0.1 µm) which, in turn, travel farther than the former. Moreover, it is demonstrated that forecast accuracy is significantly improved by taking into account ash particles density and shape; notably, spherical shape and fixed density characteristics are usually considered as default in the models.

The main need addressed in this paper is related to the lack of accurate information about the amount and size of dispersed ash particles, especially with a high degree of resolution. The latter is mainly due to the complexity and high cost of available equipment to monitor the ash fall-out phenomena. The availability of low-cost solutions enabling the utilization of such information, on a local scale, might be strategic in terms of properly feeding prediction models.

This paper investigates the possibility of performing reliable and accurate measurements of the geometric characteristics of volcanic ash using low-cost solutions.

The next sections summarize state-of-the-art methods and techniques for analyzing ash characteristics, as well as the approach proposed through this work.

### 1.1. The Analysis of the State of the Art

Different approaches have been proposed for the monitoring of volcanic eruptive activity, with particular reference to ash fallout phenomena, based on satellite platforms and terrestrial technologies (seismic instruments, radar, vision systems and laboratory equipment) [16]. In [17] an example of real-time monitoring of explosive volcanic activity to forewarn an airport is reported. The paper concerns the system deployed at the Sakurajima volcano, Japan, located 10 km from the Kagoshima City airport. The proposed monitoring system, jointly developed by Japan Air Lines and the Sakurajima Volcanological Observatory, is aimed at detecting explosive eruptions at Sakurajima Volcano, including under adverse weather and light conditions, and immediately alerting airport staff. The approach is based on the continuous monitoring of both the seismic activity and infrasonic waves produced by the volcano. By correlating these two parameters, the system is able to distinguish explosive and ash-generating eruptions from other types of volcanic activities. 

Satellite remote sensing techniques have been extensively used for volcano monitoring, particularly with the aim of identifying and tracking volcanic ash clouds [18,19,20,21,22,23,24]. Images in the visible and infrared spectra provided by geostationary satellites have been used to monitor ash clouds, making it possible to track their trajectories on a large scale [25,26]. Satellite images are typically updated every 15–30 min and are usually characterized by a spatial resolution of a few kilometers. It must be noted that the effectiveness of satellite monitoring techniques in the visible range is dramatically reduced at night, and that both visible and infrared imaging techniques may be affected by the presence of water and ice clouds at high altitudes, which can partially or totally obscure the field of view of the satellite sensors. Data provided by near-polar orbiting satellites have also been used to track volcanic ash clouds [19,27]. However, such satellite systems provide updated images of a given location once or twice a day per satellite, resulting in poor temporal resolution. National Oceanic and Atmospheric Administration (NOAA) satellites equipped with Advanced Very High-Resolution Radiometer (AVHRR) sensors, providing daily updated images and medium spatial resolution, have also been widely adopted to study volcanoes [28,29].

Among the remote sensing techniques applied to volcano monitoring, the use of ground-based microwave meteorological radar for the detection of volcanic ash clouds is widely documented in the literature [30,31,32,33,34,35,36]. The main advantages offered by this approach are its ability to operate in all weather conditions, its spatial resolution less than a few hundred meters and its update frequency, which is in the order of few minutes. Furthermore, these systems make it possible to estimate the ash volume, as well as the total mass and height of the eruptive plume. The latter parameter is of particular importance for air transport safety. Due to its potential, considerable research effort is being dedicated to improving the performance of this technology for the quantitative detection of volcanic ash [32,37].

Another consolidated approach for the monitoring of volcanic eruptions is based on the use of cameras and video surveillance systems, both in the visible and infrared spectra. Compared to visible-range cameras, images in the infrared spectrum are less affected by environmental conditions, particularly fog and clouds. Moreover, infrared cameras also make it possible to monitor the thermal evolution of volcanic phenomena. Volcanic video surveillance systems typically consist of one or more cameras. Each camera is coupled with a wireless communication interface to send data to a remote monitoring station, where images are processed and stored. Large data storage capabilities and the presence of personnel to analyze the acquired images and alert authorities in case of an emergency are typically required with such an approach. In order to overcome the need for real-time human monitoring and to optimize data storage requirements of conventional video surveillance systems, in [38], an automated volcanic video surveillance system is proposed that makes use of an infrared camera and dedicated software tools for automatic real-time image processing, framerate optimization and alerting in case of emergencies. The proposed system performs thermo-graphic analyses of the monitored volcano area and analyzes the evolution of a temperature histogram, allowing it to automatically categorize volcanic events into three classes: (i) the absence of eruptive activity, (ii) the emission of gas and (iii) lava effusion.

The approaches for volcanic ash monitoring introduced above, each with their own peculiarities, provide information on the characteristics of the ash dispersed in the plume, which is not always suitable to feed predictive models. Moreover, in most cases, these solutions are characterized by high cost, installation and maintenance difficulties and, most of all, low spatial and/or temporal resolution.

Predictive models to estimate volcanic ash dispersion require reliable information about the volcanic ash properties of the considered sample, notably, its dimensions.

Laser light scattering combined with image analysis techniques are used in automated particle size distribution analysis equipment [39]. Such instruments are usually intended for a laboratory environment rather than for real-time measurements. Particle size distribution analyzers can be used to directly estimate volcanic ash sample granulometry over a wide measurement range (10 nm ÷ 5000 µm) with very high accuracy (30 nm). Despite the excellent performance they provide, particle size distribution analyzers are better suited to offline volcanic ash granulometry analysis, due to their high cost, weight, bulk and complex sample preparation and loading procedures.

Early warning systems to promptly provide information about falling ash should be characterized by a good degree of reliability, high spatial resolution (requiring the use of several low-cost monitoring stations) and real time operation. In fact, only if the information provided to the forecasting models satisfies such requirements can reliable predictions be performed of the spatial and temporal evolution of volcanic ash clouds and areas potentially affected by ash fallout. This would make it possible to plan dedicated actions to mitigate the effects of volcanic ash fallout on human activities.

In such a context, a convenient approach could be based on the use of a network of monitoring stations distributed over the area of potential interest, equipped with a low-cost sensing system to evaluate ash properties. Such a distributed approach would allow for the real-time monitoring of ash fallout phenomena with high spatial resolution.

An early-warning, distributed monitoring system for the determination of the granulometry of volcanic ash particles and flow rate has been developed at DIEEI of the University of Catania [40]. The proposed monitoring approach is based on a network of interconnected sensing nodes. Each measurement node consists of a collector which conveys precipitated volcanic ash into a chamber. An infrared sensor array is then used to measure the temporal evolution of the ash particle volume, thus obtaining an indirect estimation of ash flow rate. A piezoelectric sensor placed inside the collector provides indirect estimations of particle sizes by measuring the impact force of the ash particles.

In [41], an improvement of the system described in [40] was presented. It concerns the use of a digital magnetometer for the purpose of better distinguishing volcanic ash particles, which exhibit paramagnetic behavior, from other types of sediments (e.g., sand), which could seriously affect the reliability of the monitoring system. Each sensing node was also equipped with meteorological sensors, providing useful information to feed forecasting models. The instrumented chamber uses a motorized movable bottom plate, making it possible to periodically remove the accumulated ash particles. The main limitation of above solutions is related to possible spurious information generated by multiple impacts of the same particle on the sensor surface, which would affect the accuracy of estimates of particle numbers and their granulometry. A similar problem arises with possible aggregates of particles. Moreover, this approach does not provide information about the dimensions of ash particles.

### 1.2. Summary of the Proposed Approach

In order to overcome the limitations of the approaches presented in the previous section, efforts have been dedicated to the development of low-cost vision systems which would enable the realization of wide sensor networks for the monitoring of volcanic ash fallout phenomena [42,43]. The idea is to exploit low-cost embedded vision systems and image processing algorithms to obtain reliable and accurate estimates of ash characteristics, with particular regard to ash dimensions.

A preliminary study of a LabVIEW vision tool which is able to perform suitable processing of digital images of samples of volcanic ash collected on a working white-plate is presented in [42]. The tool is able to extract the main geometric characteristics of each ash grain in the picture, such as its perimeter, area and the minor/major axes of its bounding rectangle. The extracted information is then saved into a dedicated file for further post-processing and/or transmission to remote data centers.

A first implementation of the image processing methodology on an embedded architecture was proposed in [43]. The tool exploits the PiCamera v2.1 camera module, equipped with an 8-megapixel Sony IMX219 image sensor [44] and a Raspberry Pi 4-based processing unit to grab and elaborate images of volcanic ash deposited on a white-plate, respectively.

With such a configuration, the achieved resolution was 24.4 µm over an 80 × 60 mm^2^ framed area, while an accuracy (in the 3σ limit) of ±7.25% was estimated for samples of 4 mm in length.

In line with aims of the SECESTA-VIASAFE project, which addresses the development of a distributed measurement system for the monitoring of volcanic ash fall-out phenomena in areas used for air traffic, in this paper, a step forward with respect to the developments described in [42,43] is proposed. In particular, the possibility of enlarging the framed area (hosting the ash samples) and improving the accuracy of the measurement system for ash size estimations is investigated.

To this end, a new, vision-based embedded system has been developed. The tool uses a new monitoring chamber and a Pi camera HQ connected to a Raspberry Pi 4-based processing unit, running a dedicated image processing algorithm.

The main novelties of this approach with respect to the work presented in [43] are as follows:

− the improvement of the vision system performance, also by using a high-resolution camera which is better able to meet the requirements of ash granulometry investigations;
a new experimental setup, including an acquisition chamber and a lighting system, which improve the working conditions, notably by illuminating the viewing plane;
new features implemented in the algorithm in order to cope with needs which emerged during the development, debugging and real-time operations;
a deep characterization of the system with respect to different particle shapes and a different set of particle characteristics, comprising perimeter, area, long axis and short axis;
a robustness analysis of particle dimensions, position in the view plane and orientation.

The main outcomes of the proposed approach are the following:

− It uses a low-cost embedded vision system for the analysis of volcanic ash, with particular regard to estimates of the dimensions of ash particles. This aspect is of fundamental importance, given the need to monitor large areas of territory which may potentially be affected by ash falls.
the possibility of easily integrating the embedded vision system into the monitoring nodes discussed in [40,41].
as a consequence of the previous outcomes, the ability to provide experts with information on the fallout of volcanic ash with high spatial resolution and in real time; this is a strategic requirement for reliable forecasts of the effects of ash fallout and to take appropriate countermeasures.

The developed methodology is presented in the next section, along with the experimental setup and the approaches adopted in the design of the vision system. The results obtained in our experimental characterization of the ash monitoring system are presented in Section 3, while tests performed with real ash samples are shown in Section 4.

## 2. The Developed Vision Embedded System

The main idea behind the proposed approach is to collect a sample of falling ash particles on a white background plate, which has to be moved inside a shielded chamber equipped with a digital camera that takes images of the ash sample. The images are then automatically processed by a dedicated algorithm implementing the image processing methodology described in Section 2.1. The algorithm runs on a Raspberry Pi-based processing unit; it serves to extract the main geometric characteristics of each detected particle.

The main specifications to be fulfilled by the system are:

− a framed area larger than 10.0 cm by 8.0 cm;
considering that the volcanic ash size of interest covers a particle range of 200 to 4000 µm, and supposing that the smallest dimension of the particle image needs be digitized with 5 pixels (for reliable recognition), the required resolution for the vision system should be better than 40.0 μm.
accuracy (in the 3σ level) regarding the system resolution in case of the smallest particles and better than 5% in case of larger ash grains.

The above specifications are not achievable with the setup adopted in [43].

### 2.1. Image Processing Algorithm

The system operation is based on a dedicated algorithm implemented in Python, using the image processing tools available through the OpenCV open-source computer vision library [45]. The algorithm estimates many geometric parameters of each particle contained in the acquired image, including its perimeter, area and the long and short axes of its bounding rectangle.

**Figure 1:**
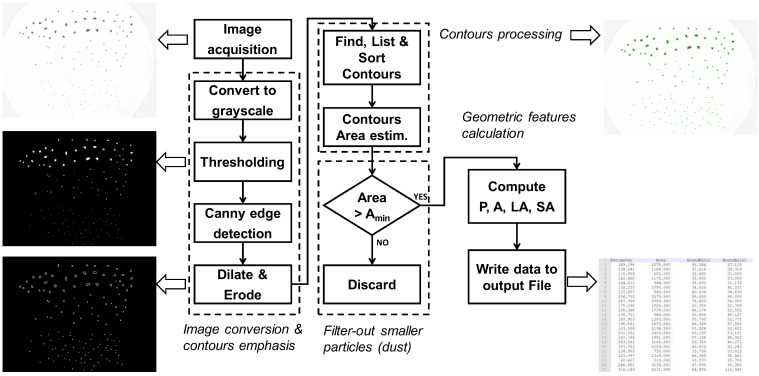
Flowchart of the image processing algorithm, together with some intermediate results generated by the system.

The sequence of operation of the image processing algorithm is represented in Figure 1, [42,43], which also shows examples of intermediate outputs generated by the system.

Basically, the images of the volcanic ash samples acquired by the camera are converted to black-and-white by applying grayscale conversion and a thresholding filter in sequence. Then, the Canny edge detection, dilate and erode filters are applied with the purpose of enhancing the particle contours, followed by specialized processing steps devoted to contour analysis.

In order to overcome the possible influence of exotic particles, i.e., dust, a filter is implemented for particles with areas smaller than a given threshold, which are discarded during the successive analysis steps. The geometric features of particles are saved as text file on a micro-SD card.

### 2.2. Experimental Setup

**Figure 2:**
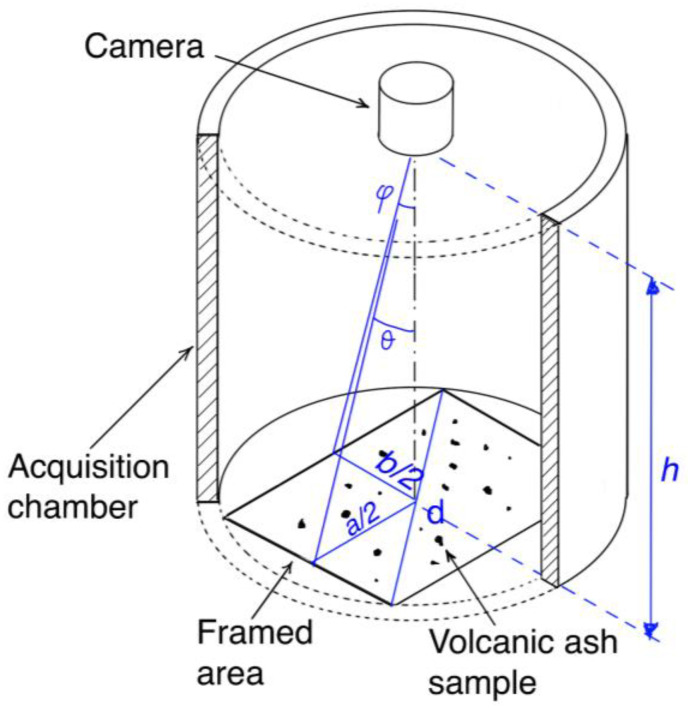
Schematic view of the experimental setup.

In this paper, the design and realization of a new prototype is investigated. The system uses a camera–lens combination that guarantees better performance with respect to a previous implementation while maintaining the low-cost characteristics. In particular, a Pi Camera HQ module is used as image sensor [46], combined with a lens with a focal length of 16 mm, a maximum aperture of f/1.4 and a C mount [47]. The Pi Camera HQ module uses a 12.3 Megapixel Sony IMX477 image sensor and 4:3 aspect ratio (4056 × 3040 pixels) in the 1/2.3″ format (7.9 mm diagonal). The different characteristics of the adopted image sensor and lens, compared to the first prototype presented in [43], require the design of a new setup. Figure 2 shows a schematic view of the experimental setup.

The camera is placed in the upper part of a light, airtight chamber, with the lens facing downward, to frame the support containing the samples of volcanic ash particles. The sealed chamber is intended to limit the influences of external light and climatic conditions on the measurement, as well as to provide mechanical support for the camera and the embedded platform running the image processing algorithm.

In order to ensure controllable and repeatable lighting conditions, a white LED illuminator was specially manufactured, positioned in the upper part of the sealed chamber and powered by a power supply located outside the chamber.

The shooting distance of the camera has been determined in such a way that the framed area, defined by the project specifications, is visible to the entire sensitive surface of the image sensor in order to optimize the resolution of the system. In order to ensure clear images, the shooting distance must be at least equal to the minimum focusing distance of the lens.

As shown in Figure 2, with simple geometric considerations, the following relationships may be deduced relating the dimensions of the framed area, *a* and *b* respectively, the shooting distance, *h*, and the horizontal and vertical angles of field of the lens, θ and ϕ respectively:

(1) $$a=2h\cdot \tan\left(\frac{\theta }{2}\right)$$

(2) $$b=2h\cdot \tan\left(\frac{\phi }{2}\right)$$

The horizontal and vertical angles of view of the lens are respectively θ=21.8° and ϕ=16.4°, while the minimum focusing distance is 20 cm. By applying (1) and (2), it can be seen that a shooting distance *h* equal to 35.0 cm corresponds to a framed area with dimensions *a* = 13.0 cm and *b* = 10.0 cm, which is compatible with the project specifications. This result allowed us to predict a nominal resolution of 32.9 µm in the worst case scenario.

If a cylindrical shape is adopted for the sealed chamber, it must have (i) a diameter at least equal to the diagonal of the framed area, so that this is entirely contained within the chamber, and (ii) a height at least equal to the shooting distance. Considering the dimensions obtained for the framed area, the minimum diameter of the sealed chamber must satisfy the following relationship:

(3) $$d\geq \sqrt{a^{2}+b^{2}}$$

By substituting the dimensions of the framed area obtained in (3), the minimum diameter for the sealed chamber is found to be equal to about 16.0 cm.

**Figure 3:**
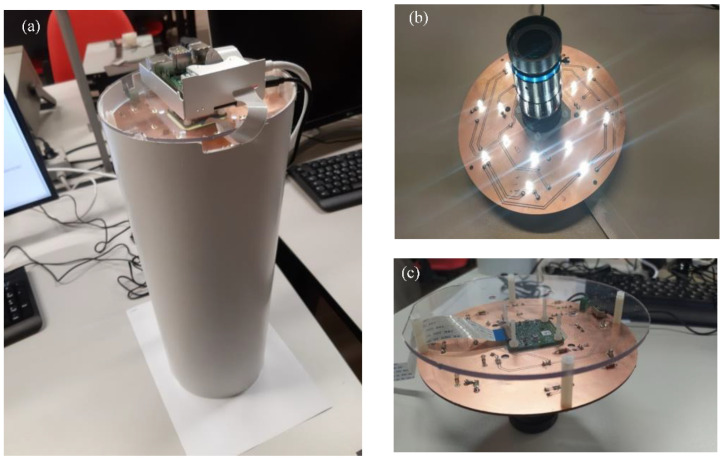
The experimental setup implementing the vision system: (**a**) the whole setup, (**b**) bottom and (**c**) top view of the lighting module assembled with the camera and lens.

In the experimental setup, the following parts have been used: a PVC sealed chamber; a dedicated PCB, housing the LED-based lighting system and acting as a mechanical support for the camera-lens assembly; and the upper part of the sealed chamber, closed with a polycarbonate cover housing the Raspberry Pi 4 board. The setup is shown in Figure 3a, while Figure 3b,c show the lower and upper parts of the LED lighting module with the camera and the lens, respectively.

The image processing algorithm shown in Figure 1 runs on a Raspberry Pi 4 model B single board computer, equipped with 8 GB RAM LPDDR4 and an ARM Cortex A72 quad-core processor at 1.5 GHz. Linux-based Raspberry Pi OS is installed as the operating system. The Raspberry Pi 4 represents a good tradeoff between computing capabilities, low power consumption, reduced size/weight and low-cost. Moreover, it can benefit from a wide selection of open-source software packages and tools.

Several command-line options have been implemented within the Python script in order to add more flexibility to its usage. These options include: (i) processing of pre-recorded digital images, (ii) saving intermediate results generated during each processing step, (iii) limiting outcomes of image processing to particle identification and counting.

To debug the system, the image processing algorithm was executed through the command line interface of the terminal emulator included in the Raspberry Pi OS distribution.

After fixing the setup and the shooting distance, tests were performed to determine the optimal exposure conditions inside the chamber. Exposure determines the amount of light that reaches the image sensor, affecting the density of the captured frame; if the light that reaches the sensor is insufficient, the final image will be very dark and the final image will be more affected by noise, causing artifacts that might be recognized by the algorithm as ash particles (false positives). Conversely, excessive exposure will produce a very clear image, in which the smallest particles will blend into the white background, resulting in the algorithm not recognizing ash particles (false negative). It is therefore evident that optimal exposure parameters are an essential requirement to ensure the efficiency of the ash particle recognition algorithm. Since the lighting conditions inside the sealed chamber can be considered very stable, thanks to the use of the lighting system and its shielding from ambient light, the parameters that influence the exposure are the aperture of the lens diaphragm, the shutter speed and the ISO sensitivity of the image sensor. The aperture has been fixed to a value that guarantees an appropriate depth of field, thus optimizing the focus of the ash particles. Therefore, the exposure can be adjusted by acting both on the shutter speed and ISO sensitivity parameters of the image sensor.

The protocol adopted to estimate the optimal exposure was the following: a set of three test images was used, each consisting of a 7 × 7 matrix of squares with dimensions of 0.2 mm, 0.4 mm and 1.0 mm. Test images were positioned inside the sealed chamber and captured by the vision tool using different combinations of shutter speed and ISO sensitivity. For each acquired image, the residual between the expected number of particles and particles recognized by the algorithm was used as the performance index to determine the optimal settings. Our analysis was performed by varying the shutter speed in the range of 1000 to 16,000 µs and the ISO sensitivity in the range of 50 to 800. The combination 2000 µs and 800 ISO achieved the best performance for all three dimensions of the considered test images.

## 3. Experimental Characterization

**Figure 4:**
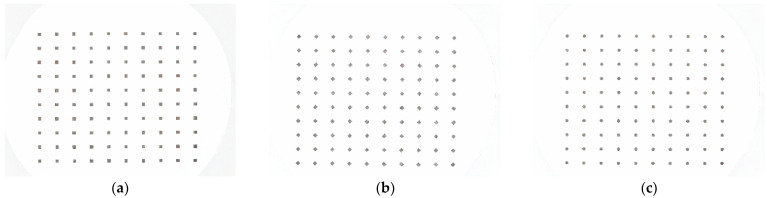
Some examples of test images used for the characterization of the vision system. Each image is a 10 × 10 matrix of (**a**) unrotated 2.00 mm squares, (**b**) 45° rotated 2.0 mm squares and (**c**) 2.0 mm circles.

An experimental characterization of the vision system has been implemented through a measurement survey based on the acquisition and processing of a set of reference printed images. Each image is composed of a matrix of particles (10 × 10) with well-defined (i) geometries (square and circle), (ii) dimensions (side or diameter: 0.2 mm, 0.4 mm, 1.0 mm, 2.0 mm and 4.0 mm) and (iii) orientations (rotation angle: 0°, 22.5°, 45.0° and 67.5°). Some examples of reference images acquired by the system are shown in Figure 4.

Printed test images have been placed inside the chamber, acquired by the camera and processed by the algorithm described in Section 2.1. The result of the processing is a text file with a 2D array structure, where each row reports the geometric quantities estimated for one particle in each image. For the purposes of the experimental characterization of the system, the considered geometric parameters are: perimeter (P), area (A), bounding rectangle long and short axes (LA, SA).

The experimental characterization of the system was carried out according to the following measurement protocol:

− for each image, ten sequential acquisitions are performed, each time removing and repositioning the image inside the vision chamber.
A MATLAB script has been developed to process data generated by the vision system. In particular, for each image, the tool calculates the mean value, $P_{i}$, and standard deviation, *σ_i_*, of each geometric parameter, estimated through the 100 particles in the image (*i* assuming different suffixes for the geometric quantities of *P*, *A*, *LA*, *SA*). The values given by the tool are in pixels.
The above estimations have been used to build a calibration diagram and to perform analyses of both the repeatability and robustness to particle position in the framed area.

**Figure 5:**
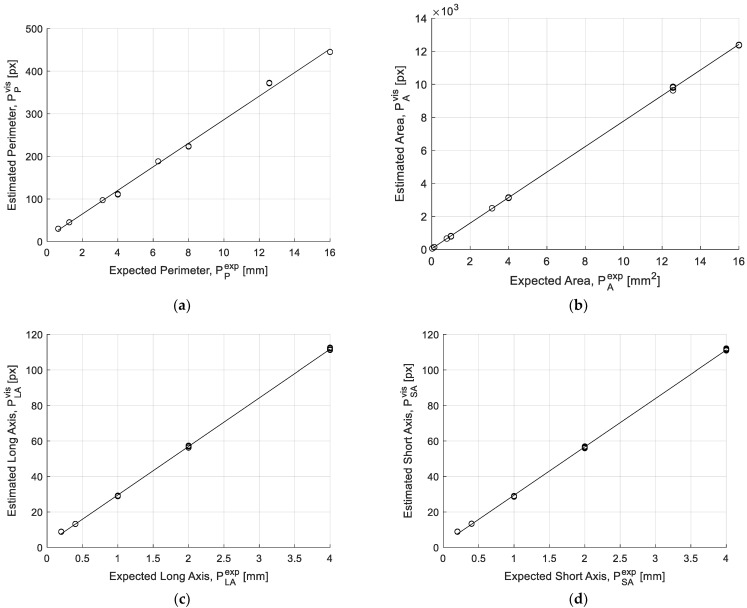
Transduction diagrams for (**a**) perimeter, (**b**) area, (**c**) bounding rectangle long and (**d**) short axes.

Figure 5 shows transduction diagrams for each of the considered geometric quantities. Such diagrams have been obtained by considering, for each image, mean values *P_i_*, estimated by the vision tool over the 100 particles, as a function of the dimensions of the particle. For the sake of clarity, it must be specified that for each particle dimension, the values of *P_i_* obtained for each acquisition (combinations of two considered shapes and 10 repetitions) are reported.

**Figure 6:**
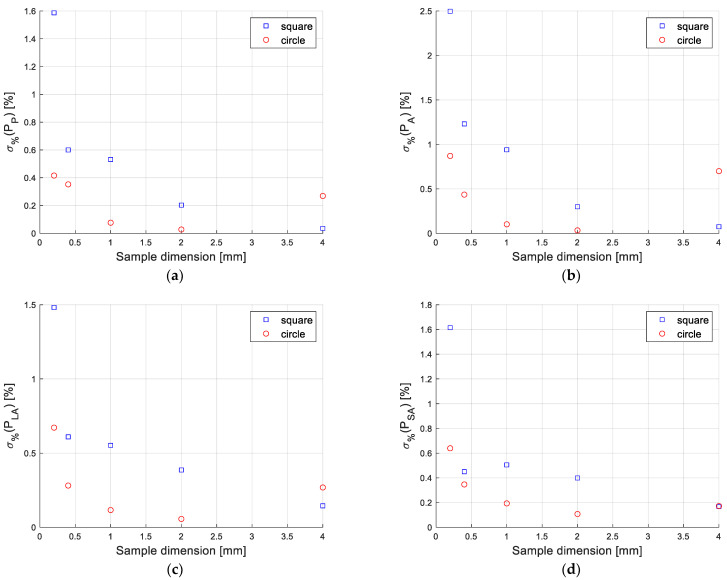
Repeatability of the system, in terms of the standard deviation, $\sigma_{\%}\left(P_{i}\right)$, of $P_{i,q}$ (*q* for 10 repetitions) for each operating condition (two considered particle shapes and dimensions): (**a**) perimeter, (**b**) area, (**c**) long and (**d**) short axes.

The obtained results highlight the small dispersion of $P_{i}$, which demonstrates the satisfactory repeatability of the system. The latter is represented in Figure 6 in terms of the relative standard deviation, $\sigma_{\%}\left(P_{i}\right)$, of $P_{i,q}$ (*q* for 10 repetitions) estimated for each operating condition (two considered shapes and different particle dimensions):

(4) $$\sigma_{\%}\left(P_{i}\right)=\frac{std\left(P_{i}\right)}{mean\left(P_{i}\right)}$$

where $std\left(P_{i}\right)$ and $mean\left(P_{i}\right)$ represent, respectively, the standard deviation and the average value of the $P_{i}$ geometric parameter, both calculated over the ten repetitions, for all considered shapes and particle dimensions.

In the worst case, a repeatability of 2.5% has been estimated.

Starting from results shown in Figure 5, the following best linear fittings have been obtained, which relate the values of *P_i_* estimated by the vision tool, $P_{i}^{Vis}$(px), and the expected values of $P_{i}^{Exp}$(mm):

(5) $${\hat{P}}_{i}^{vis}=k_{i}\cdot P_{i}^{exp}+q_{i}$$

where $k_{i}$ is the mm-to-px conversion coefficient and i=P, A, LA, SA.

In detail, the linear fitting equation and the standard deviation values of residuals between $P_{i}^{Vis}$ and model estimation, ${\hat{P}}_{i}^{Vis}$, $\sigma_{i}^{Vis}$, for each geometric feature, are the following:

(6) $${\hat{P}}_{P}^{vis}=27.67\cdot P_{P}^{exp}+9.48$$

(7) $$\sigma_{P}^{vis}=4.37\;px$$

(8) $${\hat{P}}_{A}^{vis}=773.21\cdot P_{A}^{exp}+45.71$$

(9) $$\sigma_{A}^{vis}=23.99\;px^{2}$$

(10) $${\hat{P}}_{LA}^{vis}=27.36\cdot P_{LA}^{exp}+2.21$$

(11) $$\sigma_{LA}^{vis}=0.35\;px$$

(12) $${\hat{P}}_{SA}^{vis}=27.26\cdot P_{SA}^{exp}+2.17$$

(13) $$\sigma_{SA}^{vis}=0.36\;px$$

**Figure 7:**
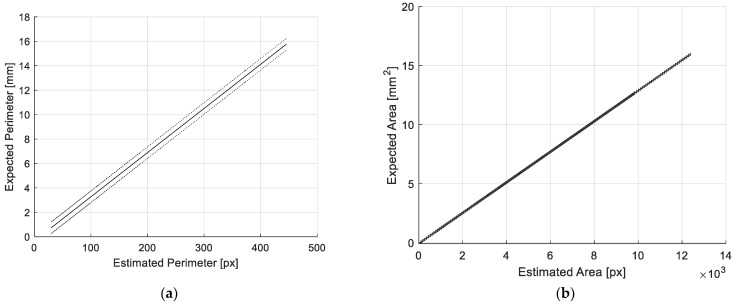

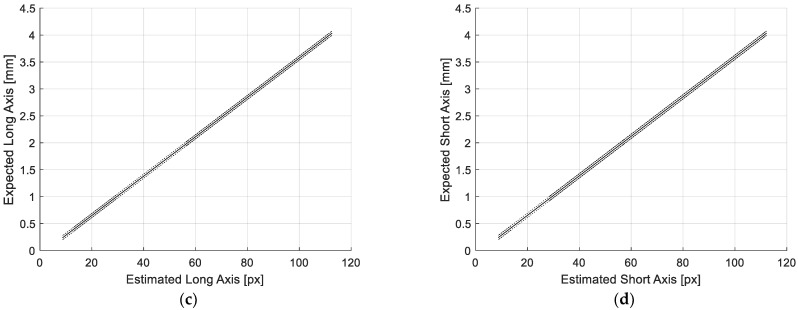
Calibration diagrams for (**a**) perimeter, (**b**) area, (**c**) long and (**d**) short axes. The uncertainty bandwidth in the 3σ limit is also shown.

The corresponding calibration diagrams are reported in Figure 7, along with their uncertainty bandwidth estimated in the 3*σ* limit.

The estimated conversion coefficients are reported in Table 1, together with the uncertainty values, $U_{i}$, for each geometric feature.

Since the main focus of the vision system is the estimation of volcanic ash particle granulometry, the average value between the long and short axis conversion coefficients could be considered as the global mm-to-px conversion coefficient of the system, which results in:

(14) $$k=27.31\;\frac{px}{mm}$$

**Figure 8:**
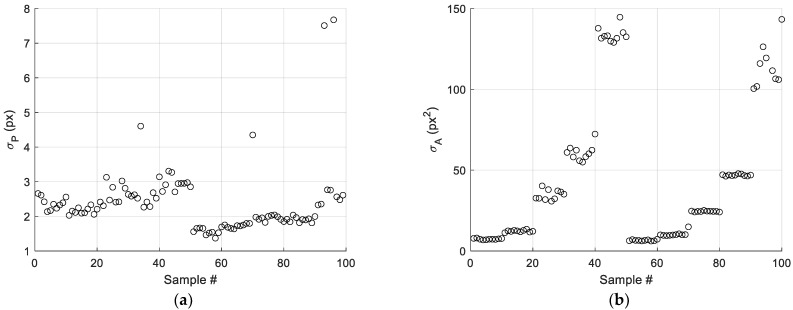

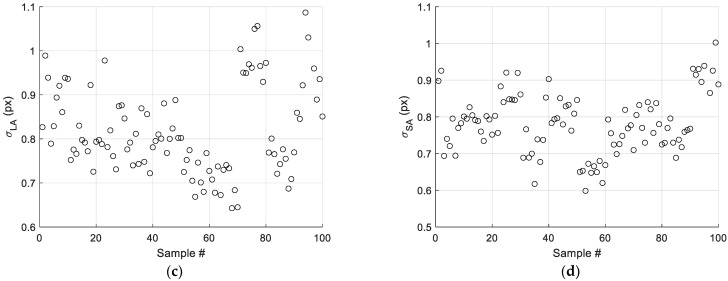
Standard deviation observed during the repeatability test over the considered particle dimensions for (**a**) perimeter, (**b**) area, (**c**) long and (**d**) short axis.

Figure 8 shows, for each geometric feature, the standard deviation of feature values obtained for particles belonging to each image, $\sigma_{i}$. By considering the plots in Figure 8c,d, it can be observed that in both cases, almost all values of $\sigma_{i}$ are below 1 pixel, confirming the robustness of the vision tool regarding particle location in the framed area.

Although the expected resolution of the system is given by 1 pixel, the contribution to the resolution given by the variability of the particle position in the framed area has also been investigated. This contribution has been estimated as the ratio between the average of $\sigma_{i}$ values, shown in Figure 8 for each parameter *P_i_*, and the conversion factor, *k_i_*:

(15) $$R_{i}=\frac{mean\left(\sigma_{i,q}\right)}{k_{i}}$$

where i=P, A, LA, SA and *q* = 1 … 100 (10 repetitions by five particle dimensions by by particle shapes).

The obtained results, presented in Table 1, show that this effect is in line with the expected nominal resolution of 32.9 µm.

The robustness of the vision tool to particle rotation has been assessed by considering test images consisting of 10 × 10 arrays of squares, having a side length of 2.0 mm and rotated by 0.0°, 22.5°, 45.0° and 67.5° (for symmetry reasons, the 90.0° rotation has not been inspected). The robustness to rotation angle has been assessed by computing the relative residuals between the estimated and nominal values of each geometric feature $P_{i}$:

(16) $$J_{i}=\frac{P_{i}^{vis}-P_{i}^{n}}{P_{i}^{n}}\cdot 100$$

where $P_{i}^{n}$ stands for the nominal value of $P_{i}$ and i=P, A, LA, SA.

**Figure 9:**
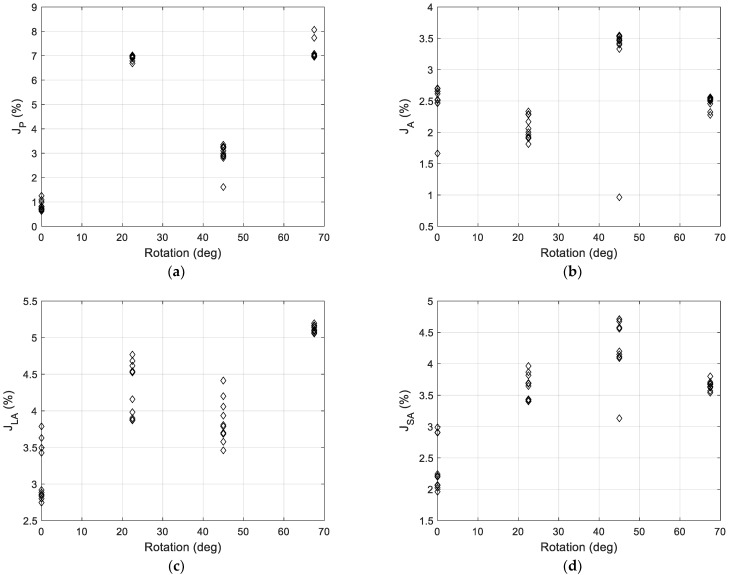
Results of the robustness test for particle rotation angle: relative residuals between the estimated and nominal values of each parameter vs. the rotation angle for (**a**) perimeter, (**b**) area, (**c**) long and (**d**) short axis.

The results of this analysis, shown in Figure 9, affirm the robustness of the system, in line with the expected system specifications.

## 4. Test with Real Ash Samples

In this section, the results provided by the developed vision tool using real samples of volcanic ash are reported. In particular, an experimental survey has been performed considering four different samples of volcanic ash belonging to the following classes of granulometry:

− G1: 0.2–1.0 mm;
G2: 1.0–4.0 mm.

Each sample has been deposited on a white support and then positioned inside the vision chamber.

**Figure 10:**
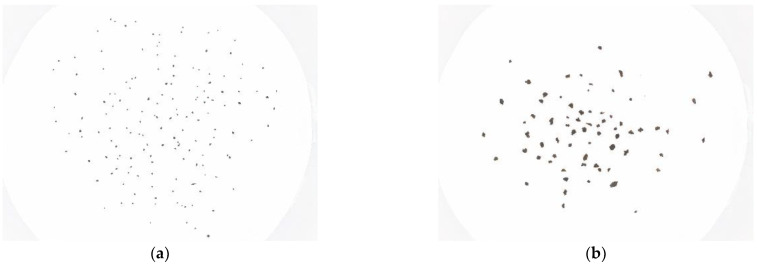
Pictures acquired by the vision system using four samples of real volcanic ash particles: (**a**) granulometry class G1, (**b**) G2.

**Figure 11:**
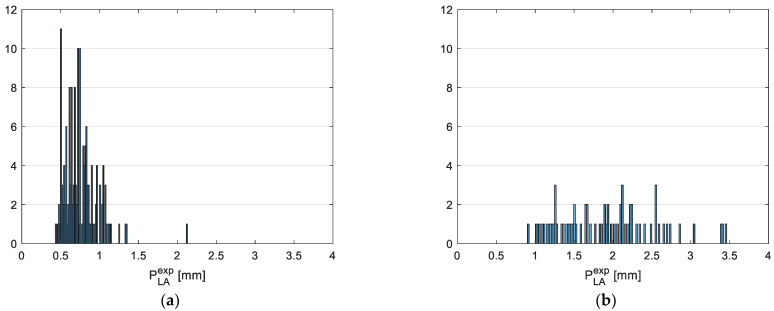

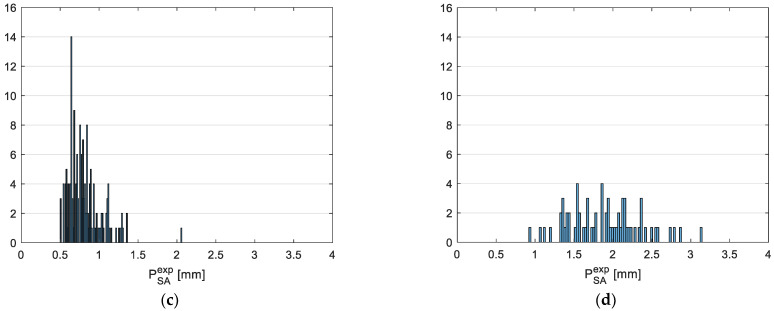
Distribution of *P_i_* characteristics over the whole analyzed sample and for all the granulometry classes considered: G1 long axis (**a**), G2 long axis (**b**), G1 short axis (**c**) and G2 short axis (**d**).

Figure 10 shows pictures acquired by the vision system for the two samples of volcanic particles. Parameters *P_i_* estimated by the tool are represented in Figure 11, in terms of their distribution over the whole sample. The obtained results highlight the coherence of estimations with the expected particle geometric characteristics. It must be noted that, being real samples, a certain level of randomness is expected in terms of the particle dimensions characterizing each of the considered samples, especially in case of very small particles, which are not always easy to correctly separate into contiguous classes. That being said, the obtained results may be used for a qualitative assessment of the system, rather than a rigorous validation like the one performed in Section 3 using reference samples.

## 5. Conclusions

In this paper, a low-cost embedded system for analyzing the main geometric characteristics of volcanic ash is presented. The proposed methodology exploits a PiCamera HQ module and a Raspberry Pi 4 model B-based processing unit to acquire and process images of ash particles precipitated in the observation area. The latter is a white support exposed to falling ashes for a defined period of time. The use of a high-resolution camera and a dedicated acquisition chamber, including a suitable illuminating system, fulfills the specifications required by real application contexts. In particular, the following values of accuracy and resolution were estimated: 40.2 µm (3σ level) and 32.9 µm, respectively. Such a result affirms the better performance of the proposed solution with respect to the one investigated in [43], considering the extension of the framed area.

The results obtained during the characterization survey demonstrated the system robustness regarding particle shape, rotation and position in the analyzed frame.

Although not yet implemented, the idea behind this approach is the realization of a moveable plate which can be exposed to falling volcanic ash particle flux for a given amount of time and then moved inside a vision chamber. This will be one of the future improvements to be implemented in the system, along with an auto-emptying procedure to remove particles from the working plate before starting a new sampling and acquisition process.

Moreover, a deep experimental survey will be performed using real samples of volcanic ash to assess the system performance with different sets of real particles.

It is worth of noting that the developed methodology could be easily extended to several application contexts requiring the analysis and characterization of other kinds of particles or grains.

## Figures and Tables

**Table 1:** Conversion coefficients, uncertainty bandwidth and resolution of the vision tool.

*P_i_*	*k_i_* (px/mm)	*U_i_*	*R_i_*
Perimeter	27.67	47.3·10^−2^ mm	87.3·10^−3^ mm
Area	27.81 *	9.3·10^−2^ mm	68.9·10^−3^ mm
Long Axis	27.36	38.2·10^−3^ mm	29.7·10^−3^ mm
Short Axis	27.26	40.2·10^−3^ mm	28.6·10^−3^ mm

* The square root value of the mm2-to-px2 coefficient has been considered for the purpose of comparison.

## Data Availability

The data presented in this study are openly available in FigShare at https://doi.org/10.6084/m9.figshare.21689126.v1.
